# The Effect of Insulin, TNFα and DHA on the Proliferation, Differentiation and Lipolysis of Preadipocytes Isolated from Large Yellow Croaker (*Pseudosciaena Crocea* R.)

**DOI:** 10.1371/journal.pone.0048069

**Published:** 2012-10-26

**Authors:** Xinxia Wang, Ming Huang, Yizhen Wang

**Affiliations:** Institute of Feed Science, Zhejiang University, Hangzhou, Zhejiang Province, People’s Republic of China; Huazhong Agricultural University, China

## Abstract

Fish final product can be affected by excessive lipid accumulation. Therefore, it is important to develop strategies to control obesity in cultivated fish to strengthen the sustainability of the aquaculture industry. As in mammals, the development of adiposity in fish depends on hormonal, cytokine and dietary factors. In this study, we investigated the proliferation and differentiation of preadipocytes isolated from the large yellow croaker and examined the effects of critical factors such as insulin, TNFα and DHA on the proliferation, differentiation and lipolysis of adipocytes. Preadipocytes were isolated by collagenase digestion, after which their proliferation was evaluated. The differentiation process was optimized by assaying glycerol-3-phosphate dehydrogenase (GPDH) activity. Oil red O staining and electron microscopy were performed to visualize the accumulated triacylglycerol. Gene transcript levels were measured using SYBR green quantitative real-time PCR. Insulin promoted preadipocytes proliferation, stimulated cell differentiation and decreased lipolysis of mature adipocytes. TNFα and DHA inhibited cell proliferation and differentiation. While TNFα stimulated mature adipocyte lipolysis, DHA showed no lipolytic effect on adipocytes. The expressions of adipose triglyceride lipase (ATGL), fatty acid synthase (FAS), lipoprotein lipase (LPL) and peroxisome proliferator-activated receptor α, γ (PPARα, PPARγ) were quantified during preadipocytes differentiation and adipocytes lipolysis to partly explain the regulation mechanisms. In summary, the results of this study indicated that although preadipocytes proliferation and the differentiation process in large yellow croaker are similar to these processes in mammals, the effects of critical factors such as insulin, TNFα and DHA on fish adipocytes development are not exactly the same. Our findings fill in the gaps in the basic data regarding the effects of critical factors on adiposity development in fish and will facilitate the further study of molecular mechanism by which these factors act in fish and the application of this knowledge to eventually control obesity in cultured species.

## Introduction

The use of high-lipid diets in aquaculture has proven to be protein saving and growth-promoting effect in some species [1], but leads to excessive fat deposition that may affect animal health and reduce harvest yields [2]. It is important to develop strategies to control excessive lipid deposition in cultivated fish to strengthen the sustainability of the aquaculture industry. As in mammals, the development of adiposity in fish arises from the hypertrophy of existing adipocytes and the proliferation and differentiation of new adipocytes, which depends on genetic, hormonal and dietary factors [3]. Therefore, in this study, we will examine the critical factors that regulate the process of adiposity development with the hope that the control of obesity in cultured species may become possible in the near future.

The development of adiposity is positively or negatively regulated by various factors. Insulin is an important anabolic hormone that can promote many cellular events in mammals, including glycogen synthesis, the regulation of amino acid transport, gene transcription and protein synthesis [4]. Insulin is required for adipocyte differentiation in mammals and birds [5], [6], and exerts an inhibitory effect on adipocyte lipolysis [7]. However, results in gilthead seabream (*Sparus aurata*) showed no effect of insulin on adipocyte lipolysis [8]. Information regarding the insulin-mediated control of fat cell proliferation and lipolysis in fish is still limited. TNFα, a cytokine, can be synthesized by and secreted from adipose tissue [9] and thus is in a position to exert a paracrine and/or autocrine role within adipose tissue. TNFα has been proven to affect many aspects of adipocyte function in mammals, ranging from adipocyte development to lipid metabolism [10]. Considering all of these diverse actions, TNFα appears to play a negative role in the development of adipose tissue. Because most of the data were generated in mammals, we were interested in determining if TNFα can exert a similar role in fish. If so, TNFα can be used to regulate lipid deposition in fish. Feeding rodents fish oil enriched in n-3 PUFA decreases adipose tissue mass and suppresses development of obesity [11]. Docosahexaenoic acid (DHA, C22:6n-3), an essential n-3 PUFA, has been reported to inhibit the proliferation of various cell types [12], [13]. The DHA-induced anti-lipogenesis and anti-lipolysis of adipose tissue has been recently described in rats [14]. Nevertheless, data measuring the ability of insulin, TNFα and DHA to manipulate adiposity development in non-mammal species, such as fish, are still scarce.

The large yellow croaker (*Pseudosciaena crocea*) is a commercially important carnivorous species, with 86,000 metric tons having been produced in China in 2010 [15]. However, the final product can be affected by excessive lipid accumulation. Therefore, a better understanding of adiposity development in this species is of value to aquaculture industries. *In vitro* cell cultures have been extensively used to elucidate the major processes involved in mammalian preadipocyte proliferation and differentiation [6]. Nevertheless, the data obtained from mammals have shown that the factors that regulate adiposity differ considerably between species [10]. An *in vitro* culture system has only been developed in three fish species [3], [16], [17]. In this case, the development of an *in* *vitro* cell culture system for the large yellow croaker is necessary to study the basic mechanisms of adipocyte biology and thereby prevent the excessive storage of lipids in this important aquaculture species. This study was therefore conducted, firstly, to define the optimal conditions for the culture of large yellow croaker preadipocytes and their differentiation into mature adipocytes, which is a preliminary step for studying the factors responsible for controlling the adipose development process; secondly, to analyze the effects of insulin, TNFα and DHA on preadipocyte proliferation, differentiation and adipocyte lipolysis; and furthermore, to elucidate the mechanisms mediating the insulin, TNFα and DHA effects, the expression of lipid-related genes during the differentiation and lipolysis of fish adipocytes was also explored.

## Results

### Preadipocytes Morphology and Proliferation

**Figure 1:**
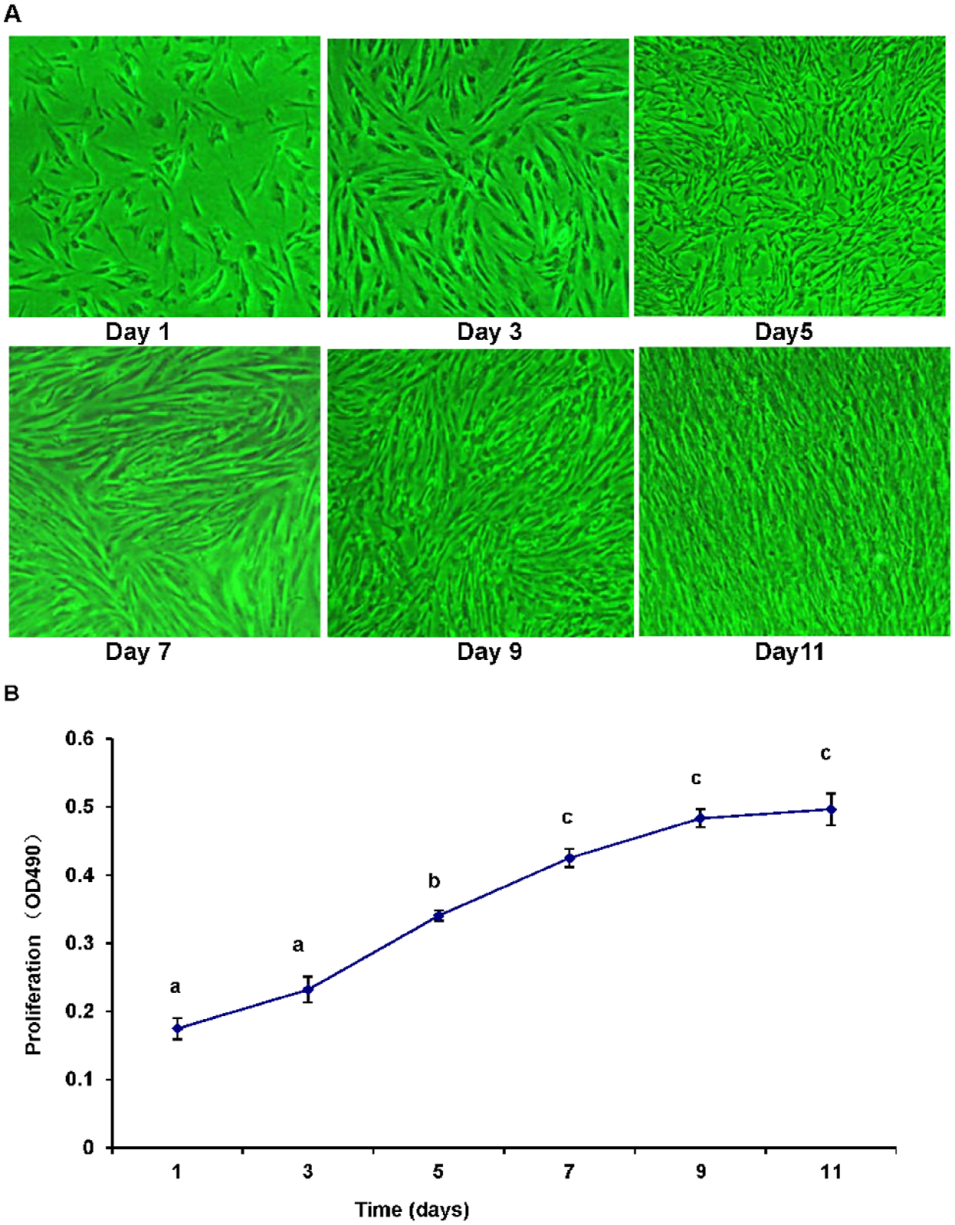
Growth of large yellow croaker preadipocytes isolated from adipose tissue at different days of growth. (A) Photomicrographs of yellow croaker preadipocytes on day 1, 3, 5, 7, 9, and 11 (× 40). (B) Proliferation profile of preadipocytes on day 1, 3, 5, 7, 9, and 11. Data are means ± SEM, n = 8. Different letters indicate significant differences at *P*<0.05.

In the present study, we first established a preadipocytes culture system using the adipose tissue of the large yellow croaker. On day 1, most of the cells were small and attached to the flask (Fig. 1A). The newly established cell culture derived from yellow croaker abdomen adipose tissue gave birth to a homogeneous population of preadipocytes with an initial fibroblast-like morphology (Fig. 1A).

To examine the proliferation of the preadipocytes in culture, MTT assay, which is based on the reduction of the tetrazolium salts by mitochondrial reductases into formazan and often used to measure cell proliferation [18], [19], was carried out in parallel to the morphological studies. As shown in Fig. 1B, the cells grew linearly until day 9 and then plateaued. This result suggested that the cells were contact-inhibited once confluence was reached, which visual observation indicated occurred at about day 9 (Fig. 1A).

### Induction of Differentiation, Morphology Observation and Gene Expression

**Figure 2:**
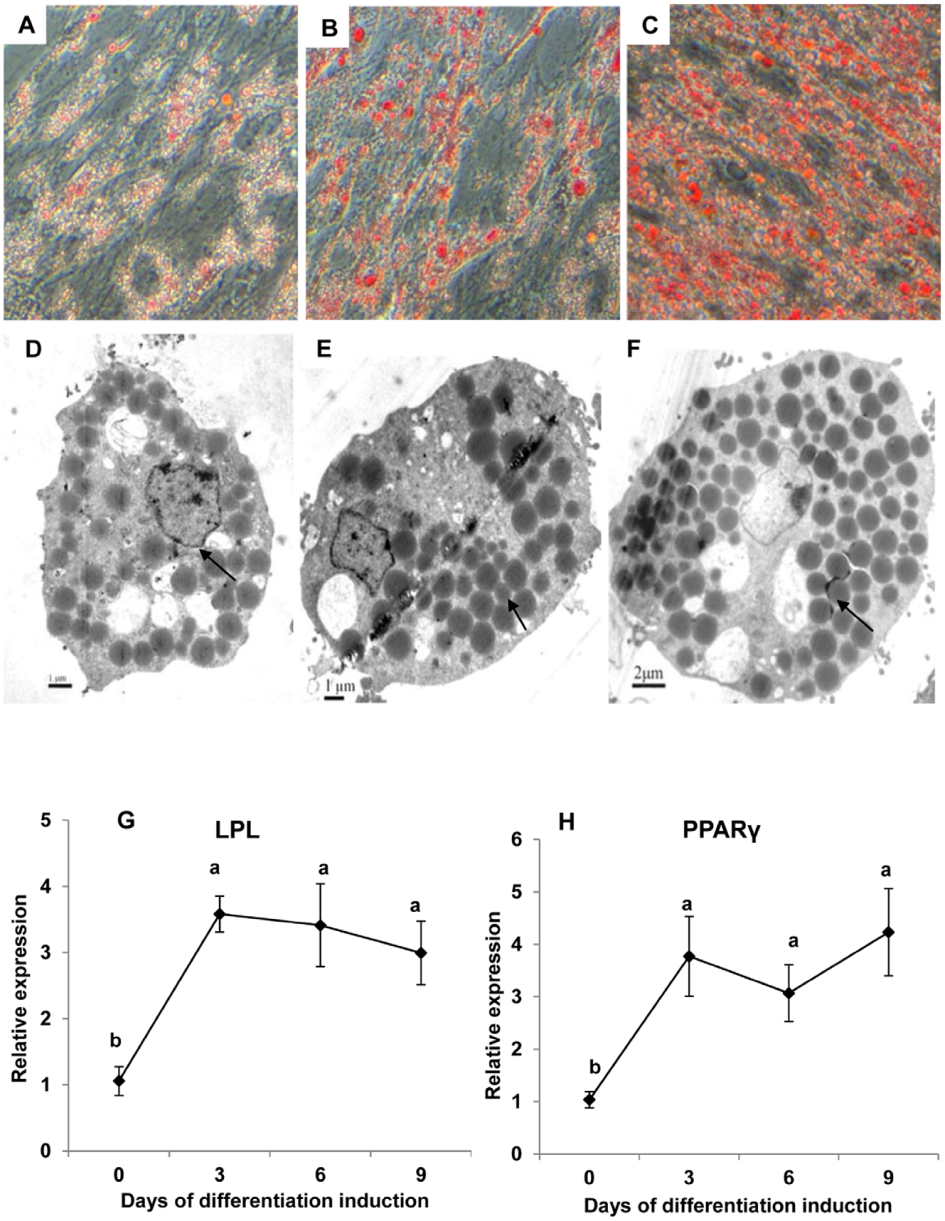
Micrographs of large yellow croaker adipocytes differentiated in culture. The cells were induced to differentiate into adipocytes and stained with oil red O at (A) day 3, (B) day 6 and (C) day 9 (×200) after induction. Electron micrographs of yellow croaker preadipocytes differentiated in culture at (D) day 3, (E) day 6 and (F) day 9 after induction. Bars: D, E = 1 µm, F = 2 µm. Arrows points to lipid droplets.

The degrees of differentiation were evaluated by GPDH activity, which is often used as an indicator of adipocyte late differentiation [6]. The GPDH activity increased from 86.21±7.22 mU/mg protein in the cells maintained in growth medium to 96.90±5.17 mU/mg protein in the cells cultured in growth medium + hormones. The GPDH activity was 103.23±8.01 mU/mg protein when the cells were incubated in growth medium + lipid mixture. The highest level of GPDH activity (133.57±8.42 mU/mg) was observed when growth medium was supplemented with both hormones and the lipid mixture (Table 1), which indicated that growth medium + hormones + lipid mixture would be the best way to induce differentiation of croaker adipocytes. To confirm the differentiation of preadipocytes, the lipids in the cells were stained with oil red O (ORO). The ORO staining on days 3, 6 and 9 showed that the cells accumulated lipid gradually (Fig. 2A, B, C). Electron microscopy showed that lipid droplets of different sizes appeared in the cytoplasm of the adipocytes 3 d after induction (Fig. 2D). The cells accumulated additional lipid droplets during the subsequent days of induction (Fig. 2E). After 9 d of induction, the lipid vacuoles became larger (bar  = 2 µm), and the cell cytoplasm was almost completely filled with lipid (Fig. 2F). The cells that were cultured in growth medium for 22 days served as a control group and showed no obvious lipid droplets in the cytoplasm (pictures not shown).

**Table 1:** GPDH activity of large yellow croaker adipocytes induced by different medium^1^.

Medium	GPDH (mU/mg protein)
Growth medium	86.21±7.22^c^
Growth medium+hormones	96.90±5.17^bc^
Growth medium +lipid mix	103.23±8.01^b^
Growth medium+hormones+lipid mix	133.57±8.42^a^

The confluent cells were induced by growth medium, growth medium + hormones (containing 10 µg/ml insulin, 0.25 µM dexamethasone, and 0.5 mM IBMX), growth medium + lipid mixture (containing 45 µg/mL cholesterol, 100 µg/mL cod liver oil fatty acids) or growth medium + hormones +lipid mixture for 6 days and evaluated by GPDH, the indicator of cell differentiation.

1 Values are mean ± SEM obtained from six wells. Different letters indicate significant differences at *P*<0.05.

By using the large yellow croaker primary adipocyte culture system thus established, the changes in LPL and PPARγ expression during adipocyte differentiation were investigated. The expression of the LPL and PPARγ genes increased during adipocyte differentiation (Fig. 2G, 2H).

### The Effect of Insulin on Cell Proliferation, Differentiation, Lipolysis and Gene Expression

**Figure 3:**
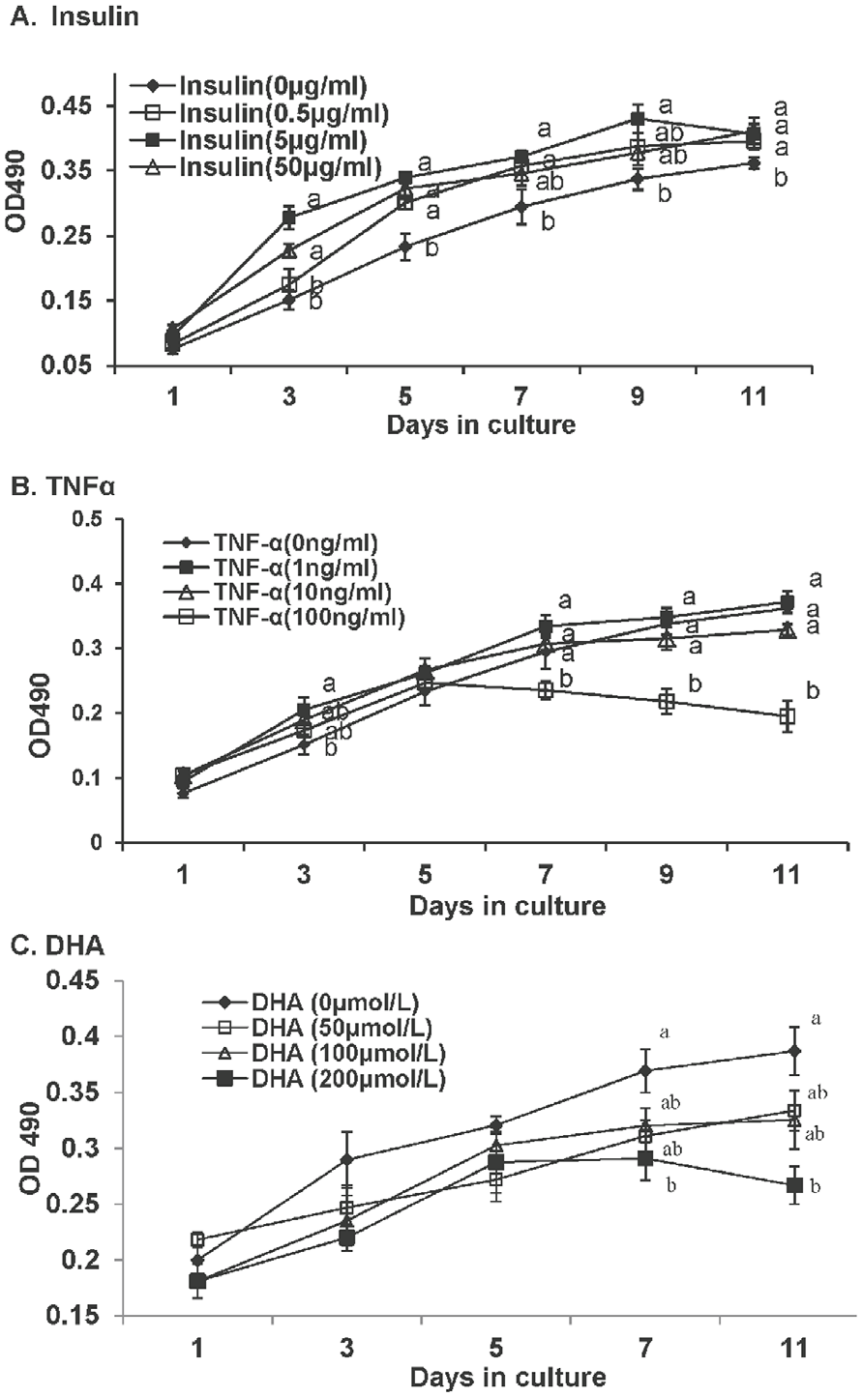
Proliferation profiles of large yellow croaker preadipocytes treated with (A) Insulin. (B) hrTNFα. (C) DHA. Cells was incubated with insulin at 0, 0.5, 5 and 50 µg/ml, TNFα at 0, 1, 10, 100 ng/ml, and DHA at 0, 50, 100 and 200 µmol/L, in separate wells from day 1 to day 11. Cells cultured in growth medium without DHA or EPA were used as control. On days 1, 3, 5, 7, 9 and 11, the cells were treated with MTT and proliferation was analyzed. Data are means ± SEM, n = 8. Different letters indicate significant differences at *P*<0.05.

To investigate the effect of insulin on preadipocytes proliferation, cells were incubated in growth medium supplemented with 0 (control), 0.5, 5 and 50 µg/ml insulin. On days 1, 3, 5, 7, 9 and 11, the cells were treated with MTT and proliferation was analyzed. As shown in Fig. 3A, preadipocyte proliferation was promoted by various concentrations of insulin.

**Figure 4:**
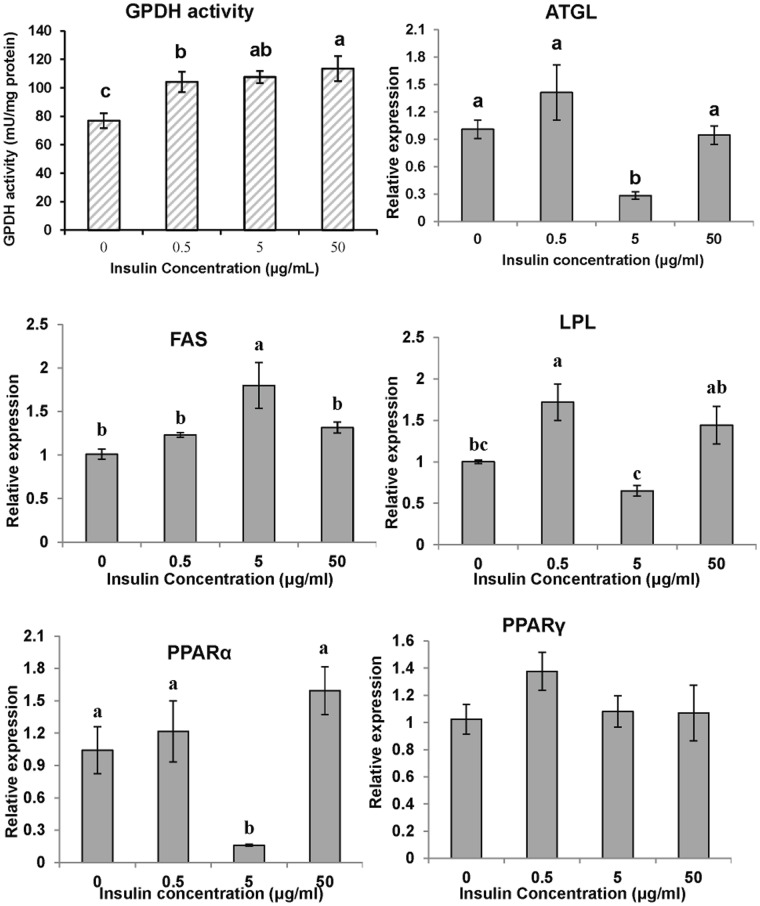
The effect of insulin on GPDH activity and gene expressions of large yellow croaker adipocytes during differentiation. The confluent cells were exposed to the differentiation medium without the lipid mixture but supplemented with 0, 0.5, 5 or 50 µg/ml insulin for 6 days and evaluated by GPDH, the indicator of cell differentiation. The gene expression levels were determined by quantitative Real-Time PCR. Data were analyzed by using 2^−ΔΔCt^ [87] and are referred to the control treatment (insulin  = 0) using β-actin as a control. Data are means ± SEM, n = 3. Different letters indicate significant differences at *P*<0.05. ATGL =  adipose triglyceride lipase, FAS = fatty acid synthase, LPL = lipoprotein lipase, PPAR  = proliferators-activated receptor α, γ.

To examine the effect of insulin on preadipocyte differentiation, cells were exposed to the differentiation media without the lipid mixture, supplemented with 0 (control), 0.5, 5 and 50 µg/ml insulin for 6 days and evaluated by GPDH activity. Compared with the control group, incubated with insulin increased the GPDH activity of adipocytes in a dose-dependent pattern (Fig. 4).

**Figure 5:**
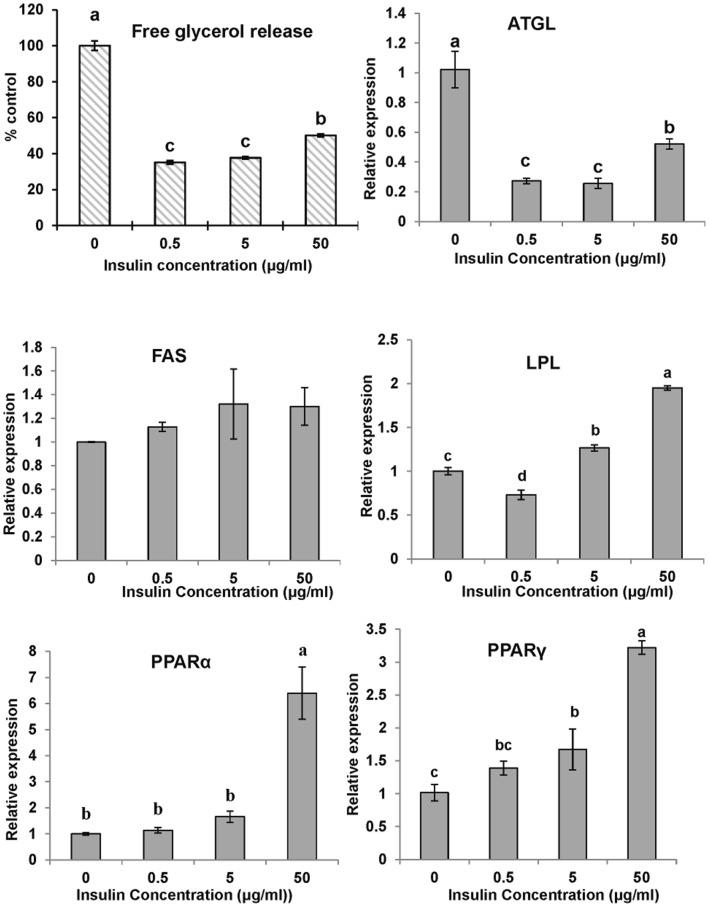
The effect of insulin on glycerol release and gene expressions of large yellow croaker adipocytes during lipolysis. The fully differentiated adipocytes were exposed to growth medium without FBS but supplemented with 0, 0.5, 5 or 50 µg/ml insulin for 24 h before the determining the glycerol concentration in the medium. The gene expression levels were determined by quantitative Real-Time PCR. Data were analyzed by using 2^−ΔΔCt^ [87] and are referred to the control treatment (insulin  = 0) using β-actin as a control. Data are means ± SEM, n = 3. Different letters indicate significant differences at *P*<0.05. ATGL =  adipose triglyceride lipase, FAS = fatty acid synthase, LPL = lipoprotein lipase, PPAR  = proliferators-activated receptor α, γ.

Because free fatty acids (FFA)-re-esterification always occurs during the incubation process [20], measuring the glycerol released into the medium offers a better estimation of the lipolytic rate than measuring FFA release. After the preadipocytes were induced to differentiate into mature adipocytes, they were treated with insulin for 24 h before determining the glycerol concentration in the medium. Insulin significantly decreased the basal lipolysis of large yellow croaker adipocytes, indicating the anti-lipolytic effect of insulin in fish (Fig. 5).

To address the molecular mechanisms of insulin-induced adipogenesis and anti-lipolysis, real-time PCR was used to analyze the expression of lipid-related genes during differentiation and the lipolysis process. Treatment with insulin (5 µg/ml) during cell differentiation significantly decreased the ATGL and PPARα levels, but increased the FAS levels in differentiated cells. A lower concentration of insulin (0.5 µg/ml) increased LPL expression, while a higher concentration showed no effect on LPL expression in cells. PPARγ expression did not change significantly during differentiation with varying concentrations of insulin (Fig. 4). Treating the cells with insulin during the cell lipolysis process decreased ATGL expression, while higher concentrations of insulin (5, 50 µg/ml) increased PPARγ and LPL expression in mature adipocytes. PPARα was significantly upregulated in the cells treated with high concentrations of insulin (50 µg/ml). However, insulin did not show any significant effects on FAS expression during adipocyte lipolysis (Fig. 5).

### The Effect of TNFα on Cell Proliferation, Differentiation, Lipolysis and Gene Expression

The proliferation of preadipocytes treated with TNFα (0, 1, 10 and 100 ng/ml) for 1, 3, 5, 7, 9, and 11 days in growth medium was evaluated by an MTT assay. The results showed that exposing the cells to higher levels of TNFα (100 ng/ml) for 3 days after seeding significantly suppressed preadipocyte proliferation (Fig. 3B). However, the continuous exposure of the cell cultures to lower levels of TNFα (1, 10 ng/ml) did not show any inhibitory effects on growth compared with the control group (Fig. 3B).

**Figure 6:**
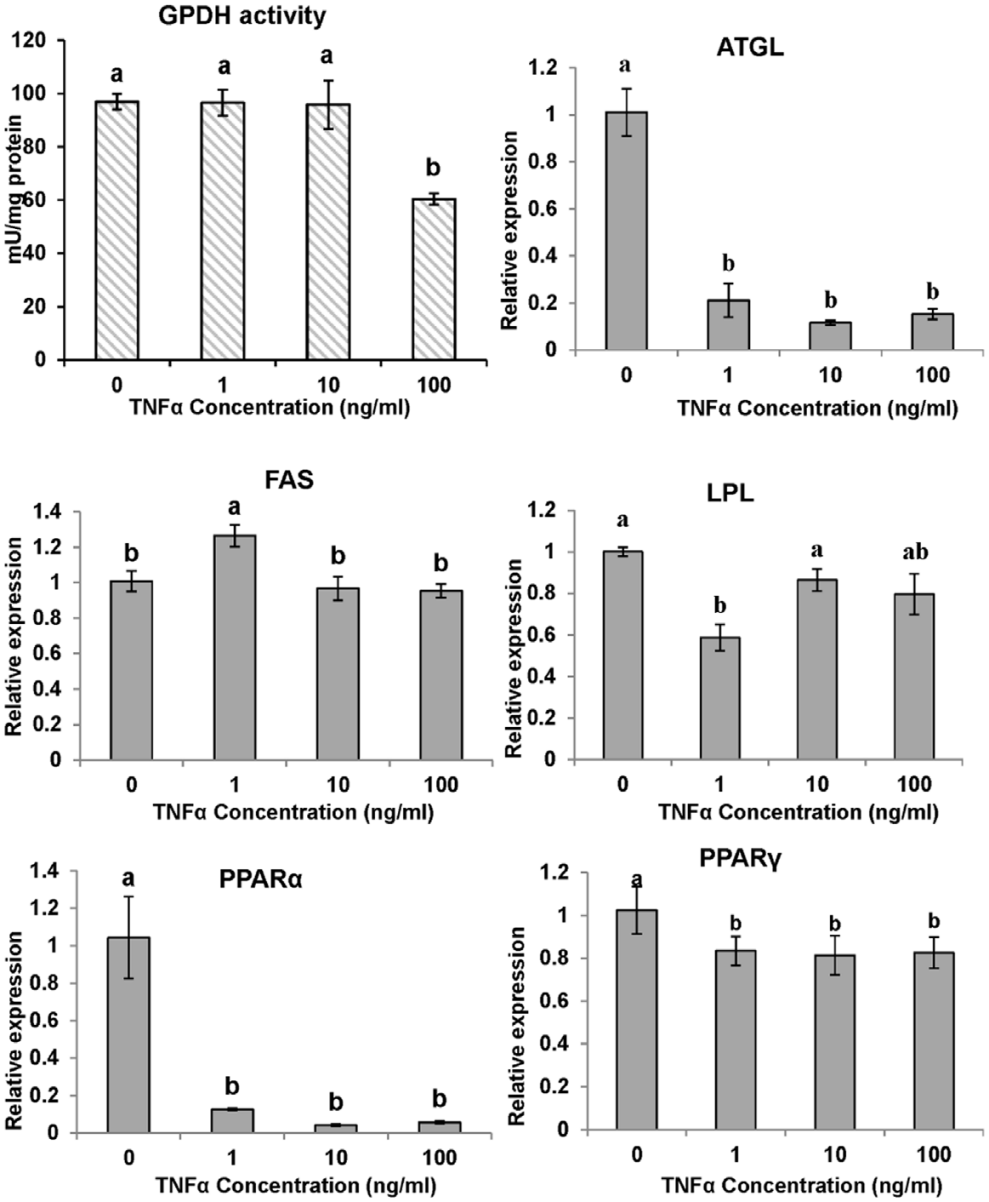
The effect of TNFα on GPDH activity and gene expression of large yellow croaker adipocytes during differentiation. The confluent cells were exposed to the differentiation medium without the lipid mixture but supplemented with 0, 1, 10 or 100 ng/ml hrTNFα for 6 days and evaluated by GPDH, the indicator of cell differentiation. The gene expression levels were determined by quantitative Real-Time PCR. Data were analyzed by using 2^−ΔΔCt^ [87] and are referred to the control treatment (TNFα = 0) using β-actin as a control. Data are means ± SEM, n = 3. Different letters indicate significant differences at *P*<0.05. ATGL =  adipose triglyceride lipase, FAS = fatty acid synthase, LPL = lipoprotein lipase, PPAR  = proliferators-activated receptor α, γ.

The GPDH activities of cells exposed to differentiation media without the lipid mixture but supplemented with 0 (control), 1, 10 or 100 ng/ml TNFα were measured. Continuous exposure to either 1 or 10 ng/ml TNFα showed no apparent inhibitory effect on cell differentiation. In contrast, the high concentration of TNFα (100 ng/ml) significantly reduced GPDH activity (*P*<0.05, Fig. 6), suggesting that high levels (100 ng/ml) of TNFα are a potent inhibitor of preadipocyte differentiation

**Figure 7:**
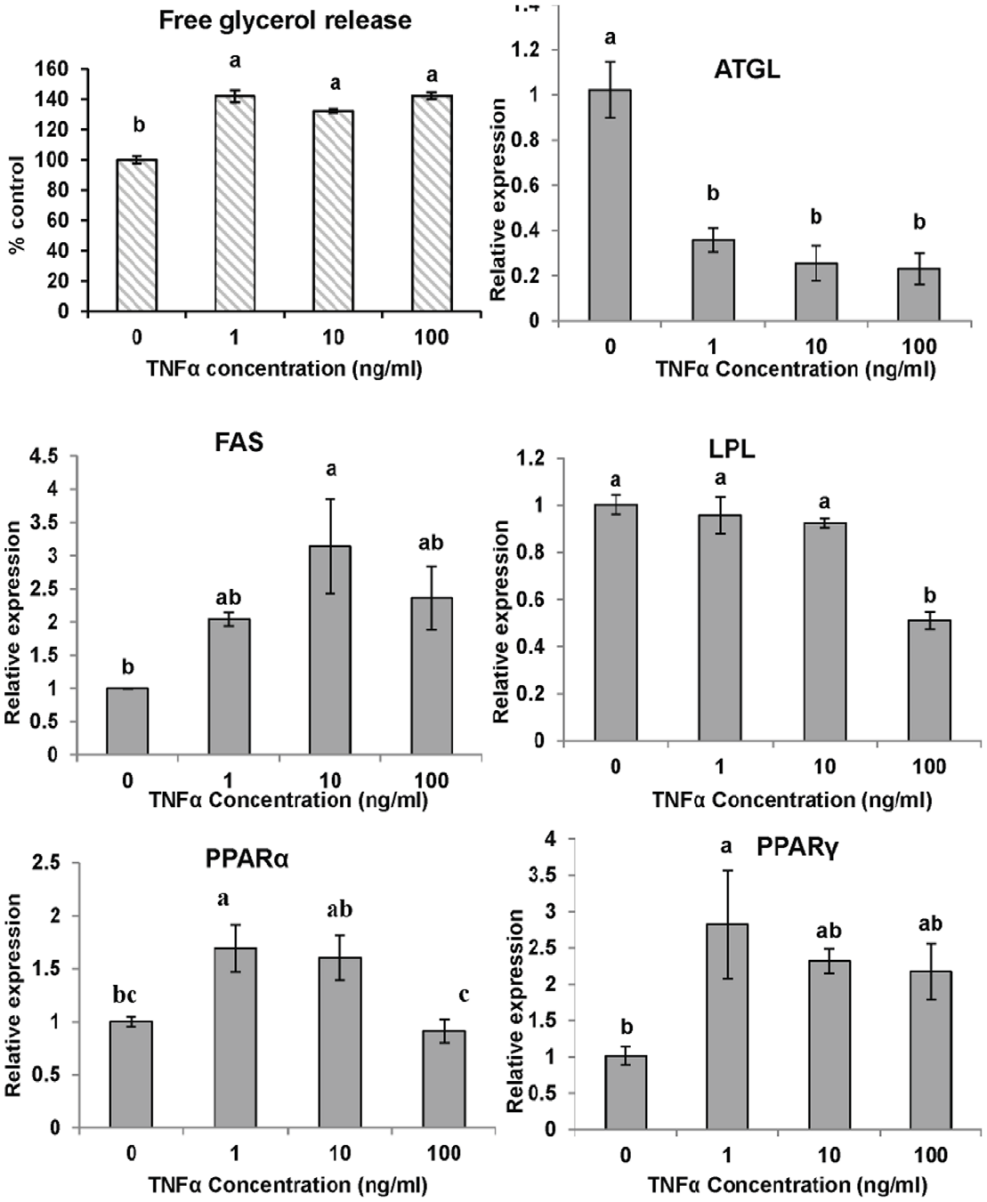
The effect of TNFα on glycerol release and gene expressions of large yellow croaker adipocytes during lipolysis. The fully differentiated adipocytes were exposed to growth medium without FBS but supplemented with 0, 1, 10 or 100 ng/ml hrTNFα for 24 h before the determining the glycerol concentration in the medium. The gene expression levels were determined by quantitative Real-Time PCR. Data were analyzed by using 2^−ΔΔCt^ [87] and are referred to the control treatment (TNFα = 0) using β-actin as a control. Data are means ± SEM, n = 3. Different letters indicate significant differences at *P*<0.05. ATGL =  adipose triglyceride lipase, FAS = fatty acid synthase, LPL = lipoprotein lipase, PPAR = proliferators-activated receptor α, γ.

TNFα (1, 10 and 100 ng/ml) stimulated glycerol release, indicating that TNFα can effectively stimulate adipocyte lipolysis in fish (Fig. 7).

In differentiating cells, the expression of ATGL, PPARα and PPARγ was inhibited by various concentrations of TNFα (Fig. 6). Although no significant effects were observed, lower LPL expression relative to the control samples was noted. FAS expression was not significantly different at the higher dose (10, 100 ng/ml). The results of the gene expression analysis of the lipolysis process are shown in Fig. 7. TNFα downregulated ATGL and LPL expression, upregulated or did not change FAS, PPARα and PPARγ expression.

### The Effect of DHA on Cell Proliferation, Differentiation, Lipolysis and Gene Expression

The addition of low dose of DHA (50 and 100 µmol/L) showed a clear tendency towards lower proliferation, although no statistical significance was observed (Fig. 3C). DHA significantly decreased preadipocyte proliferation at 200 µmol/L, suggesting that DHA had a dose-dependent inhibitory effect on cell proliferation.

**Figure 8:**
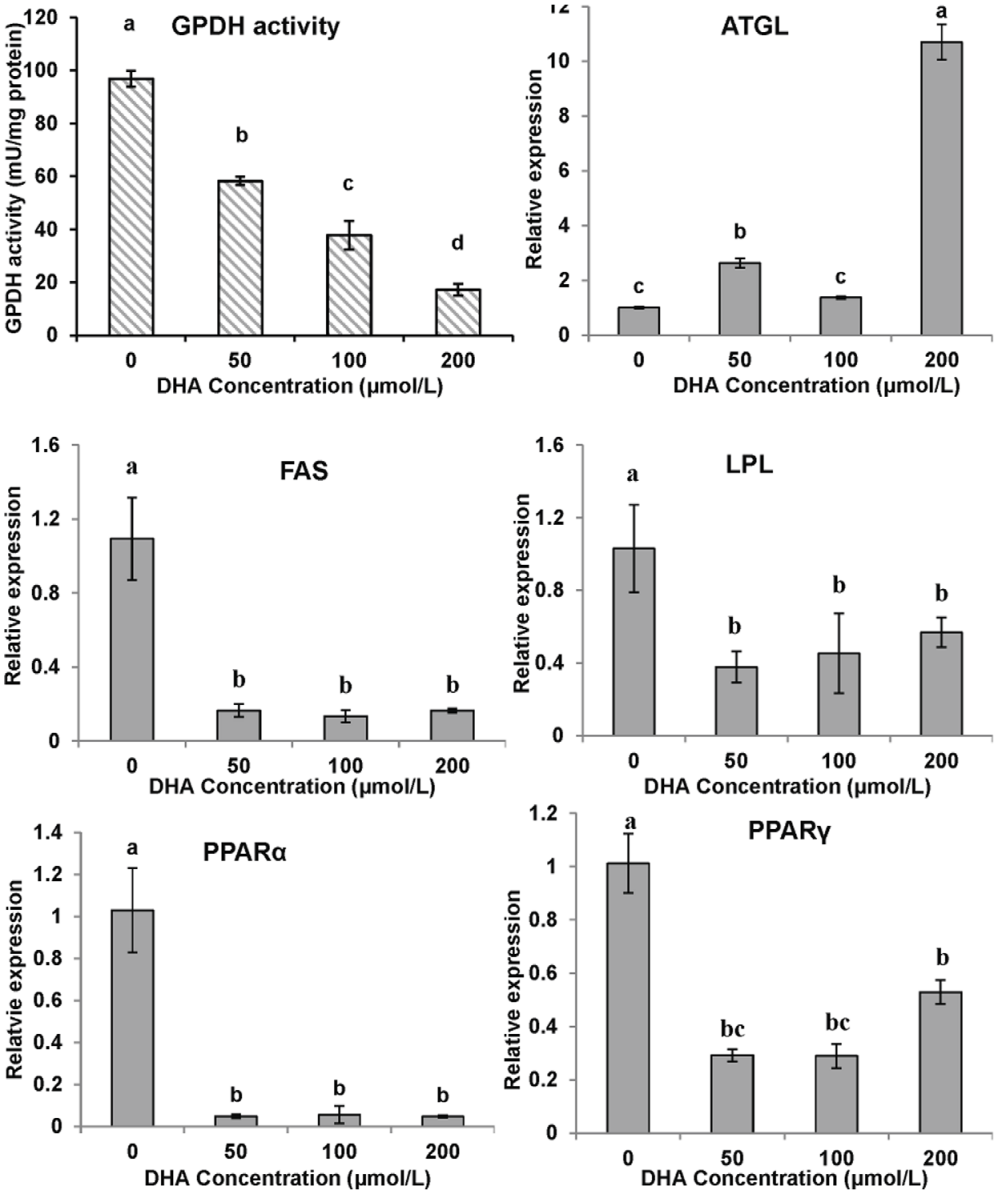
The effect of DHA on GPDH activity and gene expressions of yellow croaker adipocytes during differentiation. The confluent cells were exposed to the differentiation medium without the lipid mixture but supplemented with 0, 50, 100 or 200 µmol/L DHA for 6 days and evaluated by GPDH, the indicator of cell differentiation. The gene expression levels were determined by quantitative Real-Time PCR. Data were analyzed by using 2^−ΔΔCt^ [87] and are referred to the control treatment (DHA = 0) using β-actin as a control. Data are means ± SEM, n = 3. Different letters indicate significant differences at *P*<0.05. ATGL =  adipose triglyceride lipase, FAS = fatty acid synthase, LPL = lipoprotein lipase, PPAR = proliferators-activated receptor α, γ.

The GPDH activity of the cells exposed to differentiation media without the lipid mixture but supplemented with 0 (control), 50, 100 and 200 µmol/L DHA was significantly and dose-dependently inhibited by DHA (Fig. 8).

**Figure 9:**
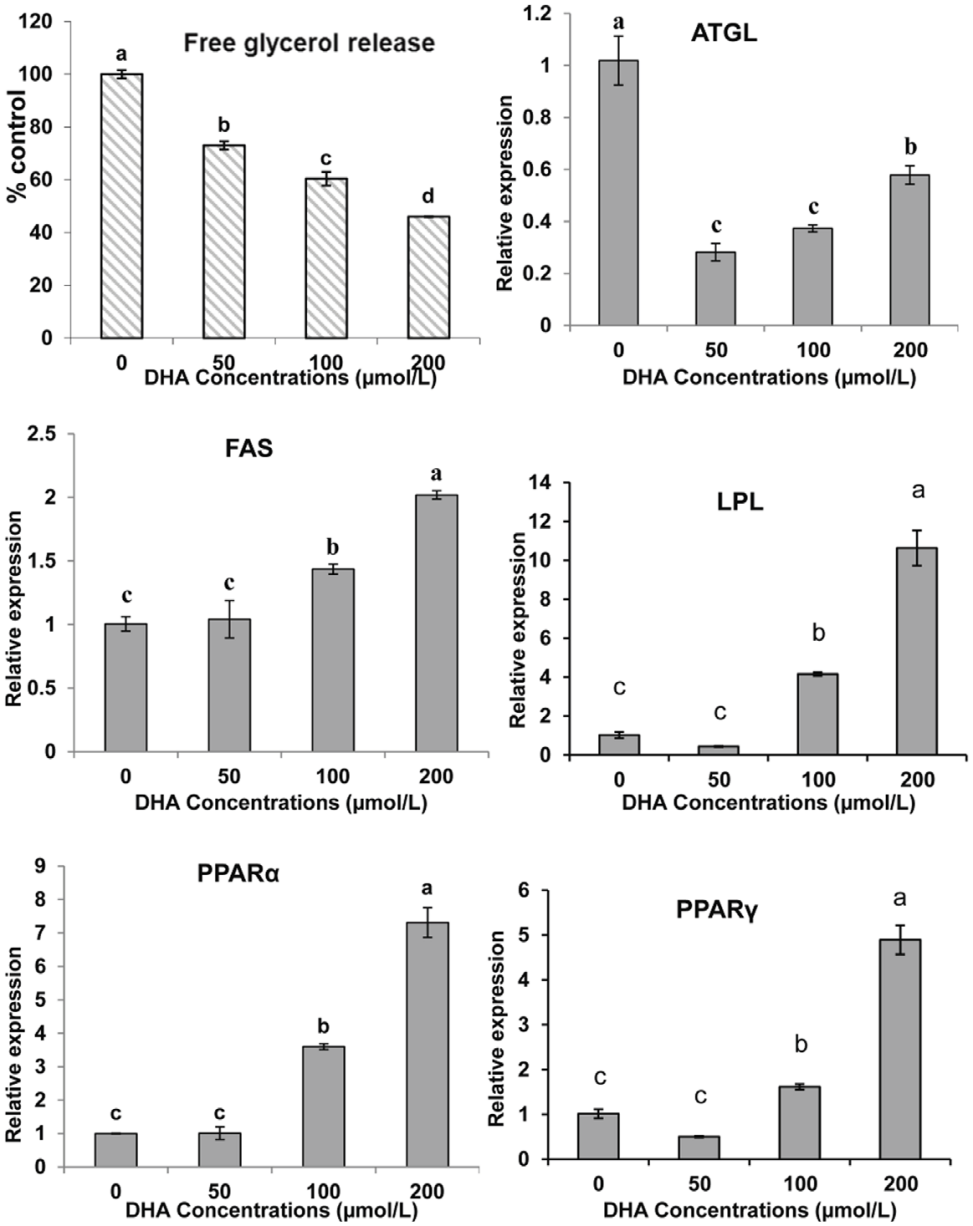
The effect of DHA on glycerol release and gene expressions of large yellow croaker adipocytes during lipolysis. The fully differentiated adipocytes were exposed to growth medium without FBS but supplemented with 0, 50, 100 or 200 µmol/L DHA for 24 h before the determining the glycerol concentration in the medium. The gene expression levels were determined by quantitative Real-Time PCR. Data were analyzed by using 2^−ΔΔCt^ [87] and are referred to the control treatment (DHA = 0) using β-actin as a control. Data are means ± SEM, n = 3. Different letters indicate significant differences at *P*<0.05. ATGL =  adipose triglyceride lipase, FAS = fatty acid synthase, LPL = lipoprotein lipase, PPAR = proliferators-activated receptor α, γ.

After 24 h of incubation, the free glycerol released into the culture medium was significantly inhibited by various concentrations of DHA (Fig. 9), suggesting that, in this system, DHA does not have the lipolytic effect described in other species.

DHA (200 µg/ml) treatment significantly increased ATGL levels but downregulated FAS, LPL, PPARα and PPARγ levels in differentiating cells at various concentrations (50, 100, 200 µmol/L) (Fig. 8). ATGL expression was downregulated during cell lipolysis, while FAS, LPL, PPARα and PPARγ expression was upregulated in mature cells.

## Discussion

In this study, we described a cell culture system designed to grow and differentiate large yellow croaker preadipocytes into adipocytes. In mammals, lipids do not seem to be a requisite for the differentiation of preadipocyte cell lines or primary preadipocytes [20], [21], [22], as hormones such as insulin, dexamethasone, and 3-isobuty-1-methylxanthine (IBMX) play a more significant role in adipocyte differentiation. In our study, hormones (insulin, dexamethasone and IBMX) or lipids alone were able to stimulate GPDH activity. However, the combination of hormones and lipids induced significantly higher GPDH activity than either component alone, suggesting that the combined application of lipids and hormones had a synergistic effect on the induction of adipocyte differentiation. The process of changing morphology from an unspecialized fibroblast-like cell type to an enlarged mature adipocyte filled with lipid droplets in the large yellow croaker is consistent with the process that occurs in other mammalian adipocyte cell lines, such as 3T3-L1 cells [23], [24] and other fish cells [25]. The lipoprotein lipase (LPL) gene, well-known as an early marker of adipocyte differentiation [26], increased at day 3 of differentiation induction, indicating its role in the early stage of large yellow croaker adipocyte differentiation. In agreement with evidence from mammals [21], [27], our results also suggest that PPARγ was induced during adipocyte differentiation. These results indicate that a cell culture system was successfully established and that the preadipocyte differentiation process in the large yellow croaker has similar properties to the same process in mammals. Using this culture system, we explored effects of hormones such as insulin, cytokines such as TNFα, and fatty acids such as DHA on fish preadipocyte proliferation, differentiation and adipocyte lipolysis *in vitro*.

Insulin promoted proliferation of large yellow croaker preadipocytes in the current study. These findings are consistent with the results from 3T3-L1 cells [28]. Evidence suggests that insulin stimulates the proliferation of various cell types through different mechanisms; for example, stimulating smooth muscle cells [29] and tumor cell lines (HT29, SW480 and MCF-7) by Akt and Erk phosphorylation, while promoting 3T3-L1 adipocyte proliferation through MAPKs [28]. The precise mechanism by which insulin stimulates fish preadipocyte proliferation has not yet been elucidated. Our data also show that insulin promoted croaker adipocyte differentiation in a dose-dependent manner, which is consistent with the report from the red sea bream [3]. In mammals, adipocyte lipogenesis can be induced by insulin, which acts through transmembrane receptors containing tyrosine kinase domains to activate various intracellular pathways [30]. The insulin-mediated mechanisms regulating adipocyte differentiation in fish are expected to be similar to those of mammals, as part of the insulin signaling pathway has been reported in rainbow trout [31]. Insulin decreased the basal lipolysis of large yellow croaker adipocytes at 0.5, 5 and 50 µg/ml, indicating its anti-lipolytic effect in fish (Fig. 5). This result is supported by studies investigating the effect of insulin on slices of adipose tissue [32] and adipocytes isolated [33] from rainbow trout. However, the results from sea bream adipocytes show that insulin have almost no effect on lipolysis [8]. A possible explanation for the differing effects may be a species-specific response to insulin [33]. The mechanism by which insulin inhibits lipolysis in mammals has been proposed to involve the reduction of cAMP levels and thus PKA activity [34], [35], which indicates that insulin exerts different actions in different pathways.

High concentrations (100 ng/ml) of TNFα inhibited large yellow croaker preadipocyte proliferation at high level, while no effect was observed at low concentrations of TNFα. Low concentrations of TNFα (0.2 nM) have been shown to stimulate the proliferation of rat adipose tissue-derived stromal-vascular cell cultures [36] and several fibroblast cell lines, including BALB/c 3T3 [37] and 3T3-L1 cells [38]. In addition, it is hypothesized that low TNFα levels may drive cells into a mitogenic program directly or utilize other mechanisms, such as keeping growth factor responsiveness [36]. However, rainbow trout preadipocytes were shown to be slightly responsive to the proliferative action of TNFα at both low (10 ng/ml) and high concentrations (100 ng/ml) [16]. Low levels of TNFα did not stimulate the proliferation of large yellow croaker preadipocytes. This finding may be linked to the origin of TNFα, as the function of TNFα is related to the species source. For example, the fact that murine TNFαhad cytotoxic effects on BALB/c 3T3 cells while human TNFαhad virtually no inhibitory action on the same cells [37] is likely the result of limited species specificity [39], [40]. Because human TNFα was used in this study, TNFα from fish species should be tested further. High levels of TNFα, which better represent septic or bacterial infection situations, may lead to negative effects on cells. Although the mechanisms are not clear, the decreased cell growth observed after treatment of rats with high levels of TNFα was not likely due to necrosis or apoptosis because no differences in lactate dehydrogenase activity were found among the treatments [36]. To date, no studies on TNFα-induced cell death of adipose tissue have been reported [41], although TNFα is well-known for its cell death inducing ability. TNFα negatively regulates adipogenesis in several adipocyte models, including 3T3-F442A [42] and human adipocyte precursor cells [43]; therefore, we were particularly interested in whether this cytokine had differential effects in our preadipocyte cultures. Kras et al. [36] reported that the inhibition of differentiation was dependent upon the level and time of exposure to TNFα. Continuous exposure to either 0.2 or 2.0 nM TNFα prevented early differentiation. Our study showed that the continuous treatment of cells with low levels of TNFα (1 and 10 ng/ml) did not significantly inhibit differentiation, but a high level of TNFα (100 ng/ml) was a potent inhibitor of preadipocyte differentiation. Similar results have been reported in 3T3-L1 [44] and rainbow trout [16]. The differences between these studies most likely arise from the origin of TNFα. García-Castillo et al. [45] demonstrated that mammalian and fish TNFα show restricted species specificity, with human (h)TNFα being unable to affect the proliferation of head-kidney cells. This finding is in sharp contrast to the strong growth-promoting effect of gilthead seabream (sb)TNFα on these cells. Conversely, sbTNFα had no cytotoxic effect on murine L929 fibroblasts, whereas hTNFα killed these cells. The mechanism through which that adipogenesis is inhibited in fish is still unclear. Previous studies have described the ability of TNFα to promote lipolysis in different mammalian cell types, such as 3T3-L1 adipocytes [46], human adipocytes [47], rat adipocytes [48] and rainbow trout adipocytes [33]. These findings support the results of the present study, which found that TNFα also promoted lipolysis in the large yellow croaker. A number of recent studies have clarified the intracellular mechanisms of lipolysis. TNFα stimulates lipolysis through multiple intracellular pathways acting on at least three separate pathways, including the inhibition of insulin receptor signaling, the inhibition of Gi-proteins and interactions with perilipin [10]. Species differences in the TNFα regulation of lipolysis have also been observed; in human cells this effect is mediated through perilipin and in rodent fat cells this effect is mediated by Gi-proteins. In fish, Albalat et al. [49] reported that TNFα stimulates lipolysis in rainbow trout adipocytes at least in part through the activation of ERK1/2 and p38 kinase. Still, more work should be completed to elucidate the TNFα mechanism of action in fish.

The antiproliferative properties of PUFAs have been shown in various types of cells, including 3T3-L1 cells [13], [50], and *in vivo* [51]. The study presented here proved that DHA exerted an anti-proliferative effect on large yellow croaker preadipocytes. However, Kim et al. [52] observed that DHA did not affect the growth of 3T3-L1 preadipocytes. The authors explained that the divergence may be due to the addition of α-tocopherol, an antioxidant that has been reported to restore cell growth after inhibition by PUFAs [53]. In this case, the anti-proliferative effect of DHA could not be excluded in study from Kim et al. DHA treatment induced apoptosis in postconfluent preadipocytes [52], but no information regarding pre-confluent preadipocytes is available. Whether the DHA-induced antiproliferative effect is due to DHA-induced apoptosis or other reasons should be studied further. In agreement with Madsen et al. [11], who proved that DHA reduces triacylglycerol accumulation during the differentiation of 3T3-L1 cells, our data indicate that DHA inhibits preadipocyte differentiation by decreasing GPDH activity in the large yellow croaker. Kim et al. [52] also found that DHA inhibits differentiation by decreasing droplet size and the percentage of lipid area. *In vivo*, perilla oil rich in n-3 PUFAs was confirmed to prevent the growth of rat visceral adipose tissue by inhibiting the late phase of adipocyte differentiation [54]. In the present study, DHA may have decreased lipid deposition by suppressing the proliferation of preadipocytes and the lipid filling of adipocytes. Very few studies have focused on the effect of DHA-stimulated lipolysis in mature adipocytes. A study from Kim et al. [52] showed that DHA stimulates the lipolysis of mature adipocytes. In contrast, no lipolysis was observed in large yellow croaker adipocytes treated with DHA for 1 to 9 h (data not shown) and, on the contrary, an antilipolytic effect was observed from the adipocyte treated with DHA for 24 h. This experiment was repeated three times independently and the same result was obtained. Because no comparable data regarding the effect of DHA-stimulated lipolysis on mature adipocytes have been reported, we postulate from our results that lipolysis may not contribute to body fat loss in the large yellow croaker. It has been suggested that body fat loss could be attributable to adipocyte deletion by apoptosis [55], which should be tested further in the large yellow croaker.

To investigate the molecular effects of insulin, TNFα and DHA on adipogenesis and lipolysis, lipid-related genes were analyzed during preadipocyte differentiation and the adipocyte lipolysis process. ATGL has recently been cloned and further characterized as a major novel TAG lipase [56], [57], [58]. Data from 3T3-L1 adipocytes [59], [60] and pig [61] show that insulin and TNFα downregulate ATGL mRNA. Information about ATGL regulation in fish is unknown. Our study defines for the first time the insulin-, TNFα- and DHA-mediated regulation of ATGL expression in fish adipocytes *in vitro*. Both anti- and pro-lipolytic factors, such as insulin and TNFα, downregulated ATGL in large yellow croaker. These results are similar to results reported in mammals [59], [60], [61]. Because insulin is the key lipogenic hormone, it follows that a lipase would be a likely target for negative regulation by insulin. The ATGL transcript was also downregulated by TNFα during both differentiation and lipolysis process, which counters the ability of TNFα to effectively promote lipolysis. ATGL is reported to possess both catabolic (lipase) and anabolic (transacylation) activities [56], and the relative contribution of these contrasting functions is unclear so far. Signaling studies from 3T3-L1 adipocytes suggest that p44/42 mitogen-activated protein kinase appears to partly mediate the negative effect of insulin but not TNFα on ATGL [60]. Whether ATGL downregulation by insulin and TNFα has the same impact on ATGL-dependent lipolysis or lipogeneis should be studied further. DHA stimulated ATGL expression in differentiating cells and downregulated ATGL expression in mature adipocytes, suggesting that DHA exerts its function differently at different stages. The sensitivity of the ATGL gene to these factors *in vitro* suggests a role for ATGL in hormone, cytokine and nutrient-induced lipogenesis and lipolysis. The mechanisms that control the ATGL-mediated process of lipogenesis and lipolysis are not understood.

Lipoprotein lipase (LPL) has been proven to play a pivotal role in regulating lipid content in mammals [62] and fish [63], and represents an early marker of adipocyte differentiation [26]. The present study revealed a marked increase in LPL expression in large yellow croaker cells during the differentiation of preadipocytes to adipocytes (Fig. 2G). Studies in fish have shown that insulin administration stimulates LPL expression in mature adipocytes [63], [64] and differentiating adipocytes [65], both of which are in accordance with our findings in present study. TNFα has been shown to downregulate LPL expression in both murine adipose tissue [66] and in 3T3-L1 adipocytes [67], which is further proved by the present study, likely via a TNFR1-dependent pathway [68]. Evidence showed that LPL mRNA was upregulated in adipose tissue fed with n-3 PUFAs [69] or perrilla oil [54]. No direct information regarding the effect of DHA on LPL expression in adipocytes was reported.

Fatty acid synthase (FAS) plays a pivotal role in de novo lipogenesis by catalyzing all of the reactions in the conversion of acetyl-CoA and malonyl-CoA to palmitate. Insulin was proven to be an effective factor in stimulating the FAS mRNA level in 3T3-L1 adipocytes [70] and human adipocytes [71]. However, it is much less clear how insulin affects the FAS transcript in fish adipocytes. Data from the current study show that FAS expression in croaker differentiating adipocytes increased significantly after treatment with 5 ug/ml insulin. Although no significant effects were observed in mature adipocytes, a clear tendency towards a higher FAS mRNA level than the control samples was noted. Insulin regulates FAS gene expression in mammalian adipocytes by modifying transcription factor binding to the insulin response element located on the promoter [72] or a specific T3 response element [73]. Despite the fact that the FAS response to insulin in large yellow croaker adipocytes is similar to the response of mammalian cells, the precise mechanisms responsible for this result are unclear. Studies have also suggested that TNFα may decrease the expression of enzymes involved in lipogenesis, specifically fatty acid synthase [74]. However, another study found that TNFα significantly increased the levels of FAS in rat hepatocytes [75]. TNFα significantly reduced the enzymatic activity of FAS, but the gene expression of FAS was unchanged [76]. TNFα did not downregulate FAS expression in either the preadipocyte differentiation or adipocyte lipolysis processes observed in this study. Because no result comparable to the effect of TNFα on FAS in fish adipocytes has been reported, a possible explanation for the variation between fish and mammals is the existence of species-specific differences.

PPARα is known to have anti-obesity effects [77] and is involved in the regulation of energy balance through fat catabolism [78]. It is paradoxical that insulin, a lipogenic hormone, activates PPARα through the phosphorylation of an AF-1 domain via the MAP kinase pathway [79]. In the present study, the PPARα level did not increase in differentiating adipocytes, while insulin stimulated PPARα expression in mature large yellow croaker adipocytes, findings that are in accordance with the above study. The TNFα-mediated downregulation of the PPARα transcript during preadipocyte differentiation and tendency of PPARα to increase during adipocyte lipolysis may be due to different roles of PPARα in lipid metabolism during various cellular conditions. PUFAs have been reported to induce the activation of PPARα, stimulate β-oxidation and reduce plasma triacylglycerol levels in rodents [11]. DHA also activates PPARα [80], which supports our finding that PPARα was upregulated by DHA in mature adipocyte. However, the sharp decrease in PPARα induced by DHA treatment during preadipocyte differentiation needs to be studied further.

It is widely accepted that insulin can activate PPARγ *in vivo* and *in vitro* [79]. In this study, we showed that insulin activates PPARγ transcription in mature adipocytes, a finding that is consistent with the results from human adipocytes. However, In contrast to the results in mammals, insulin shows no effect on PPARγ expression during cell differentiation in the large yellow croaker. Insulin showed no effect on the levels of human adipocyte PPARγ2 mRNA when the treatment lasted longer than 3 h, but can maintain higher PPARγ1 levels for quite a long time [81]. Although two isoforms of PPARγ (PPARγ short and PPARγ long) have also been reported to play different roles in salmon adipocyte differentiation [25], no additional information regarding PPARγ isoforms has been reported for other fish species. The above fact allows us to speculate that if two PPARγ isoforms exist in the large yellow croaker, then the PPARγ measured in the present study must be the “PPARγ2 mRNA” that has no lasting response to insulin. However, the time course study revealed that large yellow croaker PPARγ shows a response consistent with that observed in mammalian cells during differentiation (Fig. 2F). This effect can possibly be explained by the presence of the lipid mixture, which can serve as ligands to strongly activate PPARγ [82] in the time course study, while no lipid mixture was included in the insulin experiment. The significant increase in PPARγ expression during cell differentiation in the time course study may largely be due to the action of the lipid mixture or a synergistic effect of the lipid mixture and insulin. The PPARγ levels decreased in response to TNFα-induced cell differentiation, which means that TNFα partially inhibits differentiation by suppressing PPARγ expression in the same situation as mammals. However, PPARγ has a tendency to increase during TNFα-induced lipolysis, which the mammalian counters results showing that TNFα exerts a lipolytic effect by downregulating the PPARγ transcript [10]. It is still possible that after starvation and 24 h lipolysis, the cells can be stimulated by the free fatty acids secreted into the medium to take up and re-esterify the lipids to support cellular survival. A time course study investigating gene expression should examine time points from 1 to 24 h to determine if changes are occurring during TNFα incubation with fish adipocytes. DHA was also reported to be an activator and ligand of PPARγ [11]. In this case, DHA should stimulate differentiation. However, many studies [52], including the present one, have shown that DHA inhibits adipocyte differentiation. Taking these into account, the mechanism underlying the DHA-induced adipocyte response must be more complex than we had imagined.

In summary, the data reported in this study established an *in vitro* fish preadipocyte culture system as a useful tool for studying the nutritional and hormonal regulation of lipid metabolism in adipocytes. The results of this study indicated that although preadipocyte proliferation and differentiation in the large yellow croaker are similar to the same processes in mammals, the effects of critical factors such as insulin, TNFα and DHA on fish adipocyte development are not exactly the same. Data from mammals or other species should be applied to fish with caution. To our knowledge, this is the first time that the effects of insulin, TNFα and DHA have been evaluated in the process of fish adipocyte development. This study completes the gaps in our knowledge of the effects of insulin, TNFα and DHA on adiposity development in fish and will facilitate the further study of the molecular mechanisms through which these factors act in fish and the application of this knowledge to the eventual control of obesity in cultured species.

## Materials and Methods

### Ethics Statement

All procedures were approved by the Committee on Animal Care and Use and Committee on the Ethics of Animal Experiments of Zhejiang University.

### Establishment of Cell Culture System

#### Cell preparation and culture conditions

Large yellow croaker (*Pseudosciaena crocea* R.) between 500 and 800 g were obtained from a commercial farm in Xiangshan Port, China. The fish were sacrificed by a sharp blow to the head. The adipose tissue on the wall of abdomen was carefully excised from six fish by sterile dissection. The tissue was washed three times with HBSS and minced into small pieces. The fragments were digested in 0.2% Type II collagenase (Invitrogen Corporation, Carlsbad, CA, USA) in HBSS (1 g tissue/5 mL HBSS) at 25°C for 1 h under gentle shaking at a speed of 60 rpm. The resulting cell suspension was then centrifuged at 700×g for 10 min. The floating mature adipocytes and digestion medium were removed and the cell pellet was treated with erythrocyte lysing buffer (0.154 M NH_4_Cl, 10 mM KHCO_3_, and 0.1 mM sodium EDTA) for 10 min at 25°C. After washing twice, the cells were resuspended in a growth medium containing Dulbecco’s modified Eagle medium/HamF12 (DMEM/F12, Invitrogen Corporation, Carlsbad, CA, USA), 15% fetal bovine serum (FBS), 15 mM HEPES, 365 mg/L L-glutanmine, 100 U/mL pencillin and 100 µg/mL streptomycin. The cells were counted and plated (day 0) in cell-culture flasks (Corning incorporated, MA, USA) precoated with rat tail tendon collagen Type I (5 µg/cm^2^) (Shengyou Biotechnolgy Co Ltd., Hangzhou, China) at a density of approximately 5×10^4^ cells/cm^2^ in the medium described above. The cells were incubated at 28°C with 5% CO_2_. On the next day, the cells were extensively washed with growth medium to remove the unattached cells. The morphology of preadipocytes was observed using light microscopy, after which the cells were photographed.

#### Cell proliferation and induction of differentiation

Cell proliferation was assessed by 3-(4,5-dimethylthiazolyl-2-yl)-2,5-diphenyltetrazolium bromide (MTT) spectrophotometric assay methods [19]. Briefly, on the selected days (1, 3, 5, 7, 9, and 11), the cells were incubated in 96-well plates for 4 h at 28°C in growth medium with a final concentration of 0.5 mg/ml MTT. After washing with PBS, the cells in each well were treated with 150 µL dimethyl sulfoxide (DMSO) to fully dissolve the emerging formazan, and its absorbance was measured at 490 nm in a spectrophotometer.

Cell differentiation was induced following the method of Bouraoui et al. [16] with slight modifications. To optimize the adipose differentiation condition, the preadipocytes reaching confluence were induced to differentiate by means of growth medium + hormones (10 µg/mL insulin, 0.5 mM IBMX, 0.25 µM dexamethasone), growth medium + lipid mixture (10 µl/mL; corresponding to 45 µg/mL cholesterol, 100 µg/mL cod liver oil fatty acid (methyl esters) and growth medium + hormones + lipid mixture. The extent of cell differentiation was assessed by quantifying Glycerol-3-phosphate dehydrogenase (GPDH, EC 1.1.1.8). GPDH activity is an enzyme that catalyzes the reversible redox conversion of dihydroxyacetone phosphate to glycerol 3-phosphate, which is often used as an indicator of adipocyte late differentiation [6]. The insulin, IBMX, dexamethasone and lipid mixture were obtained from Sigma-Aldrich (St. Louis, MO).

#### Assay of GPDH activity

The GPDH activity was determined using the protocol of Sottile and Seuwen [83] with slight modification. Cells were grown and induced to differentiation with or without insulin, TNFα or DHA in 96-well plates. At day 6 after induction, cells were washed with PBS and an ice-cold homogenization solution was added (20 mM Tris-HCl, 1 mM EDTA, and 1 mM β-mercaptoethanol, pH 7.3). Cells were then stored at -20°C overnight. The next day, frozen plates were taken out and thawed at room temperature. After mechanical processing of samples with a gauge needle, the assay mixture was added to each well (0.1 M triethanolamine, 2.5 mM EDTA, 0.1 mM β-mercaptoethanol, and 334 µM NADH, pH 7.7) and plates were incubated for 10 min at 30°C. The reaction was started by adding 4 mM dihydroxyacetone phosphate. GPDH activity was measured spectrophotometrically at 340 nm. The protein content of cell cultures was determined by the Bradford method. Results were expressed as mU/mg protein (1U = 1 µmol NADH/min).

#### Oil red O staining and electron microscopy

To visualize the accumulated triacylglycerol in the adipocytes on days 3, 6 and 9 after differentiation, the cells were stained with ORO according to Ramírez-Zacarías et al. [84]. The changes in adipocyte morphology were observed using an inverted microscope and subsequently photographed.

On days 3, 6 and 9 after differentiation, the cells were harvested and prepared for electron microscopy using the protocol described by [25]. Ultrathin sections (50 µm) were prepared and observed with TEM of Model JEM-1230 electron microscope (JEOL Ltd., Tokyo, Japan).

### The Effects of DHA, Insulin and TNFα on Cell Proliferation, Differentiation and Lipolysis

To test the effects of DHA, insulin and TNFα on proliferation, cells were incubated with DHA (50,100 and 200 µmol/L), insulin (0.5, 5, 50 µg/ml), or TNFα (1, 10, 100 ng/ml) in separate wells. The cells cultured in growth medium without DHA, insulin or TNFα were used as a control. One the selected days (1, 3, 5, 7, 9 and 11) after incubation, cell proliferation was assessed by MTT as described above. Control and experimental treatments were conducted in eight replicates.

To assess the effects of DHA, insulin and TNFα on adipocyte differentiation, the GPDH activities and gene expressions in the cells exposed to differentiation medium without the lipid mixture but supplemented with DHA (0, 50,100 and 200 µmol/L), insulin (0, 0.5, 5, 50 µg/ml), or TNFα (0, 1, 10, 100 ng/ml), were measured. Control and experimental treatments were conducted in triplicate.

To investigate the effects of DHA, insulin and TNFα on adipocyte lipolysis, the glycerol contents in medium were measured after the cells were exposed to growth medium without FBS but supplemented with DHA (0, 50,100 and 200 µmol/L), insulin (0, 0.5, 5, 50 µg/ml), or TNFα (0, 1, 10, 100 ng/ml) for 24 h. The gene expressions in cells were also evaluated at the same time. Control and experimental treatments were conducted in triplicate.

### Glycerol Measurement

Mature adipocytes cultured in 6-well plates were treated with various concentrations of insulin, TNFα or DHA for 24 h. After the treatment, medium in each well was collected for measurement of glycerol concentration (as an index of lipolysis) using Free Glycerol Determination Kit (Sigma, Saint Louis, Missouri, USA) according to the manufacture’s protocol. The kit utilizes a spectrophotometric method with glycerokinase and glycerol phosphate dehydrogenase. The increase in absorbance at 540 nm is directly proportional to the free glycerol concentration of the sample.

### RNA Extraction and Quantification of Gene Transcripts

Cells for gene expression studies were thoroughly washed in PBS and collected for RNA extraction. Total RNA was extracted from the adipocytes using Trizol Reagent (Invitrogen Life Technologies, Carlsbad, CA) according to the manufacturer’s instructions. Two micrograms of total RNA were reverse-transcribed into cDNA using the M-MuLV reverse transcriptase kit (Fermentas, EU, Glen Burnie, Maryland, USA) according to the manufacturer’s instruction.

The gene transcripts levels were measured using a SYBR® Premix Ex Taq™ Kit (Takara Biotechnology Co. Ltd, Otsu, Shiga, Japan) in the ABI StepOnePlus™ Real-Time PCR System (Applied Biosystems, Foster City, CA, USA) according to the method described by Wang et al. [85]. The ATGL, FAS and LPL primers were designed from the partial yellow croaker sequences available in Genebank (**HQ916211, JN561160** and **JN247445**, respectively) using Primer premier software v5.0 (PREMIER Biosoft Internatianal, Canada) The beta-actin, PPARα and PPARγ gene primers were synthesized according to Zhao et al. [86]. The following primers were used:

ATGL-F:5′CCATGCATCCGTCCTTCAACC3′.

ATGL-R:5′GAGATCCCTAACCGCCCACT3′ (103 bp).

FAS-F: 5′ACTCCTATGTGGCAGCATAGAC3′.

FAS-R: 5′GTTTCAGCCTCAGACTCTTTGCC3′ (73 bp).

LPL-F: 5′AGACTCCATCTTCTCCTCC3′,

LPL-R: 5′TGACTTGACAAAGACTCCATC3′ (121 bp);

PPARγ-F: 5′CAGTGGCAGCGTTAAACATCG 3′.

PPARγ-R: 5′GAAGAAACCCTTACAGCCCTCA3′ (98 bp);

PPARα-F: 5′GTCAAGCAGATCCACGAAGCC 3′.

PPARα-R: 5′TGGTCTTTCCAGTGAGTATGAGCC3′ (82 bp);

β-actin-F: 5′GACCTGACAGACTACCTCATG 3′.

β-actin-R: 5′ AGTTGAAGGTGGTCTCGTGGA3′ (87 bp).

### Data Analysis

All experimental data are presented as mean ± SE and were analyzed using one way analysis of variance (ANOVA). Statistical tests were performed using the statistical program SPSS 16.0 (SPSS Inc, Chicago, IL, USA).
